# The impact of empowerment theory-based health education on Alzheimer’s disease informal caregivers: a randomized controlled trial

**DOI:** 10.3389/fpubh.2024.1393823

**Published:** 2024-08-27

**Authors:** Xiaofeng Liu, Shurui Wang, Lirong Wei, Yun Liu, Jiping Bian, Shen Wang, Xian Du

**Affiliations:** ^1^Department of General Surgery, Taihe Hospital, Hubei University of Medicine, Shiyan, Hubei, China; ^2^Graduate School of Tianjin University of Traditional Chinese Medicine, Tianjin, China; ^3^Peking Union Medical College, Beijing, China; ^4^Tianjin First Central Hospital, Tianjin, China

**Keywords:** Alzheimer’s disease, caregivers, empowerment, dementia disease knowledge, education

## Abstract

**Background:**

There is a lack of evidence regarding the effectiveness of empowerment healthy education for caregivers of Alzheimer’s patients.

**Objective:**

To explore the effectiveness of the intervention of health education guided by empowerment theory on dementia knowledge, caregiving readiness, positive caregiving emotions, anxiety, and depression in informal Alzheimer’s disease caregivers.

**Design:**

A single-blinded, randomized controlled trial.

**Setting:**

A teaching hospital in Tianjin, China.

**Participants:**

Eighty caregivers of Alzheimer’s disease patients.

**Methods:**

Participants were recruited from the hospital and randomly assigned to either experimental or control group. The experimental group underwent a 12-weeks, one-to-one intervention of six session lasting 45–60 min each. The control group received conventional health education. Outcome measures included dementia knowledge, caregiver readiness (primary outcomes), positive caregiving emotions, anxiety, and depression (secondary outcomes).

**Results:**

After 12 weeks, the intervention group exhibited significantly higher levels of dementia knowledge, caregiver readiness, and positive caregiving emotions compared with the control group. Furthermore, levels of hospitalization-related anxiety and depression were lower in the intervention group. All study results of this study showed statistically significant differences (*p* < 0.05).

**Discussion:**

Empowerment theory-based health education appears to be an effective intervention for improving caregiver and readiness to care for caregivers of Alzheimer’s disease individuals. The intervention may help reduce caregivers’ anxiety and depression levels.

## 1 Background

Alzheimer’s disease (AD) is highly prevalent among older adults and is characterized by irreversible and progressive symptoms (1). These include cognitive dysfunction, decreased ability to perform daily self-care, and psycho-behavioral symptoms as the manifestations (2). The disease can be classified into different syndromic groups such as amnestic AD, posterior cortical atrophy (PCA), and logogenic variant primary progressive aphasia (lvPPA) (3–5). Currently, the exact pathogenesis of AD remains unclear, making its prevention and treatment challenging (6).

The high incidence of AD and significant burden have become global issues (7). Studies indicate that AD patients require long-term supervision by medical personnel and caregivers, which imposes a heavy burden on society, the healthcare system, and also the patients’ family and caregivers (8). However, due to limited medical resources and substantial economic burdens, most AD patients are cared for at home or within their communities. Consequently, family members often become informal caregivers, experiencing considerable stress and burden (9).

Informal caregivers are defined as non-professional, non-volunteer, non-social workers who live with the patient and provide the majority of caregiving tasks for the longest period. This group includes the patient’s spouse, siblings, children, and other relatives (10–12). Some researchers consider a primary caregiver to be someone who provides care for at least 4 h per day (13). AD patients heavily rely on their caregivers for physical, cognitive, and emotional support, presenting significant challenges for family caregivers in terms of their caregiving abilities and psychological well-being (14).

Several studies report that family members who become caregivers for the first time often feel unprepared and lack the necessary knowledge about the disease to effectively care for the patient (15). These first-time caregivers also lack proper guidance in meeting the physical, cognitive, and emotional needs of AD patients (16–18). They frequently experience fatigue and reduced stamina (19). Additionally, negative emotions such as anxiety and depression are common among informal caregivers due to their generally low level of awareness about Alzheimer’s disease (20–22). Surveys have shown that family caregivers often do not receive adequate medical support, assistance, and caregiving information. This lack of support leads to low levels of caregiving readiness, which in turn, increases the stress and burden on caregivers (23). Inadequate preparation and insufficient disease-related knowledge also raise the risk of rehospitalization due improper caregiving behaviors (24, 25). Furthermore, the health and living conditions of family caregivers directly impact the quality of life and prognosis of patients with dementia (26).

Given this context, it is crucial for healthcare professionals to actively promote primary caregiver preparedness and disease-related knowledge to improve health outcomes of both AD patients and their informal caregivers. Research reported that empowerment education could be an effective method. Inspired by Paulo Freire, Empowerment education is a unique approach that promotes active participation and control among learners, aiming to shift power dynamics and enhance individuals’ capacity to act on their own behalf. This contrasts with conventional education interventions, which focus on addressing specific learning gaps or behavioral issues through targeted and structured teaching methods. While empowerment education fosters self-efficacy and collaborative learning, other interventions often employ individualized instruction to meet specific needs such as behavioral modifications or social and emotional skill enhancement (27, 28). In nursing, empowerment education differs from conventional health education must fulfill the following four conditions (29): (i) Effective communication between medical professionals and participants (30); (ii) Care measures tailored to individual needs, conditions, and expectations of the empowered person (30); (iii) Acquisition of the necessary knowledge base for caregiving, improvement in caregiving and problem-solving skills, and increased confidence; and (iv) Active participation as the core of empowerment enabling caregivers to improve their abilities and beliefs in caregiving through the intervention process (31). Literature reviews reveal that empowerment education has been widely applied to various contexts including glycemic control behaviors in patients with prediabetes (32), interventions in young and middle-aged cardiac patients (33), cancer patients (34), subsequent pregnancies after cesarean sections (35), caregivers of children with precocious heart disease (36), and individuals diagnosed with Alzheimer’s disease (37). However, there is still a lack of studies on interventions for informal caregivers of AD patients. This study aims to investigate the role of empowerment education for informal caregivers of patients with amnestic Alzheimer’s disease. It examines the effects on caregivers’ knowledge of dementia, caregiving readiness, positive feelings, and negative emotions.

## 2 Methods

### 2.1 Study design

This study, conducted from April 2021 to February 2022 was a prospective, single-blinded, parallel-group, randomized controlled trial. The intervention group received 12 weeks of empowerment health education along with usual care, while the control group received only usual care. Outcomes were measured in three points: before the intervention (baseline, T0), at the first follow-up (4 weeks after intervention, T1), and at the second follow-up (12 weeks, T2).

### 2.2 Participant recruitment

The study site was conducted at a memory disorders clinic of a tertiary care hospital in Tianjin, China. Participants were family caregivers of people with dementia, selected based on specific inclusion and exclusion criteria. The inclusion criteria: (a) 18 years or older; (b) Family caregiver of an individual with a confirmed medical diagnosis of dementia who has been residing in the community; and (c) Providing care for at least 3 months, with the family caregiver defined as an unpaid individual who has a significant personal relationship with the person with dementia. Exclusion criteria: (a) History of mental disorders; (b) Presence of serious or chronic diseases such as cancer or cardiovascular disease; and (c) Participation in any empowering intervention, cognitive therapy, or structured psychosocial intervention within the 6 months prior to recruitment. After applying these criteria, a research assistant explained the study’s purpose, procedure, potential benefits, and possible risks to the participants. Those who agreed to participate signed an informed consent form.

### 2.3 Randomization

The informal caregivers were randomly assigned to two groups using a computerized random number method. Each participant was assigned a number from 1 to 80 and then randomly divided into two groups labeled with 1 or 2 using SSPS 21.0. In this study, group 1 was the intervention group, while group 2 was the control group. To avoid contamination, the intervention and control groups were scheduled at different times.

### 2.4 Blinding

Blinding of the participants and the nursing researcher (QW) who delivered the intervention was not possible due to the nature of the intervention. However, the research assistant, who collected all outcome data was blinded to the group allocations.

### 2.5 Sample size

The sample size calculation used the formula for comparing the means of two independent samples (38), where σ represents the standard deviation and δ represents the mean difference. In this case, σ = 5.66 and δ=6.41 . $Z_{\alpha }=1.96$ and $Z_{\beta }=1.64$ were determined based on related research by Wang et al. (39), which used the same dementia knowledge level scale to evaluate the primary outcomes. Substituting these parameters into the sample size calculation formula, the estimated sample size was 66 participants (33 per group). Accounting for the natural attrition rate of 20% (40), the study ultimately recruited a total of 80 patients (40 per group).

### 2.6 Interventions

Two different health education approaches were applied to the control group and intervention group, respectively. The control group received conventional health education on dementia care, which focuses on treating the patient’s disease, with healthcare providers taking the lead and patients passively complying with treatments. This model aims to increase patient compliance through active disease management by healthcare workers.

In contrast, the intervention group received dementia care based on empowerment theory and self-management principles. This approach emphasizes the patient’s internal experiences as a form of psychological empowerment, involving mutual participation and equal authority between patients and healthcare providers. It aims to promote behavioral changes by encouraging patients to actively engage in health decision-making and by enhancing their self-management abilities. The comparison between conventional and empowerment education is summarized in Table 1.

**Table 1:** Comparison between education applied in each group.

Feature	Control group	Intervention group
Focus of care	Treating the patient’s disease	Participant’s internal experiences (psychological empowerment)
Role of healthcare providers	Primary leaders	Equal participants
Role of participants	Passive compliance	Active engagement
Management approach	Healthcare workers manage diseases actively	Enhance participants self-management abilities

#### 2.6.1 Control group (conventional health education on dementia care)

The control group received conventional health education, which covered topics such as AD symptoms, daily dietary guidance, sleep guidance, exercise guidance, medication guidance, home-safety guidance, outpatient medication guidance, and follow-up schedules. This program was delivered by a nurse with 3 years of work experience in dementia care. After the initial education session, participants received regular follow-ups via phone call or WeChat every 2 weeks, with each session lasting 20–30 min. To ensure consistent support, all participants were also provided with free real-time (24-h support) guidance and Q&A.

#### 2.6.2 Intervention group (empowerment education on dementia care)

**Figure 1:**
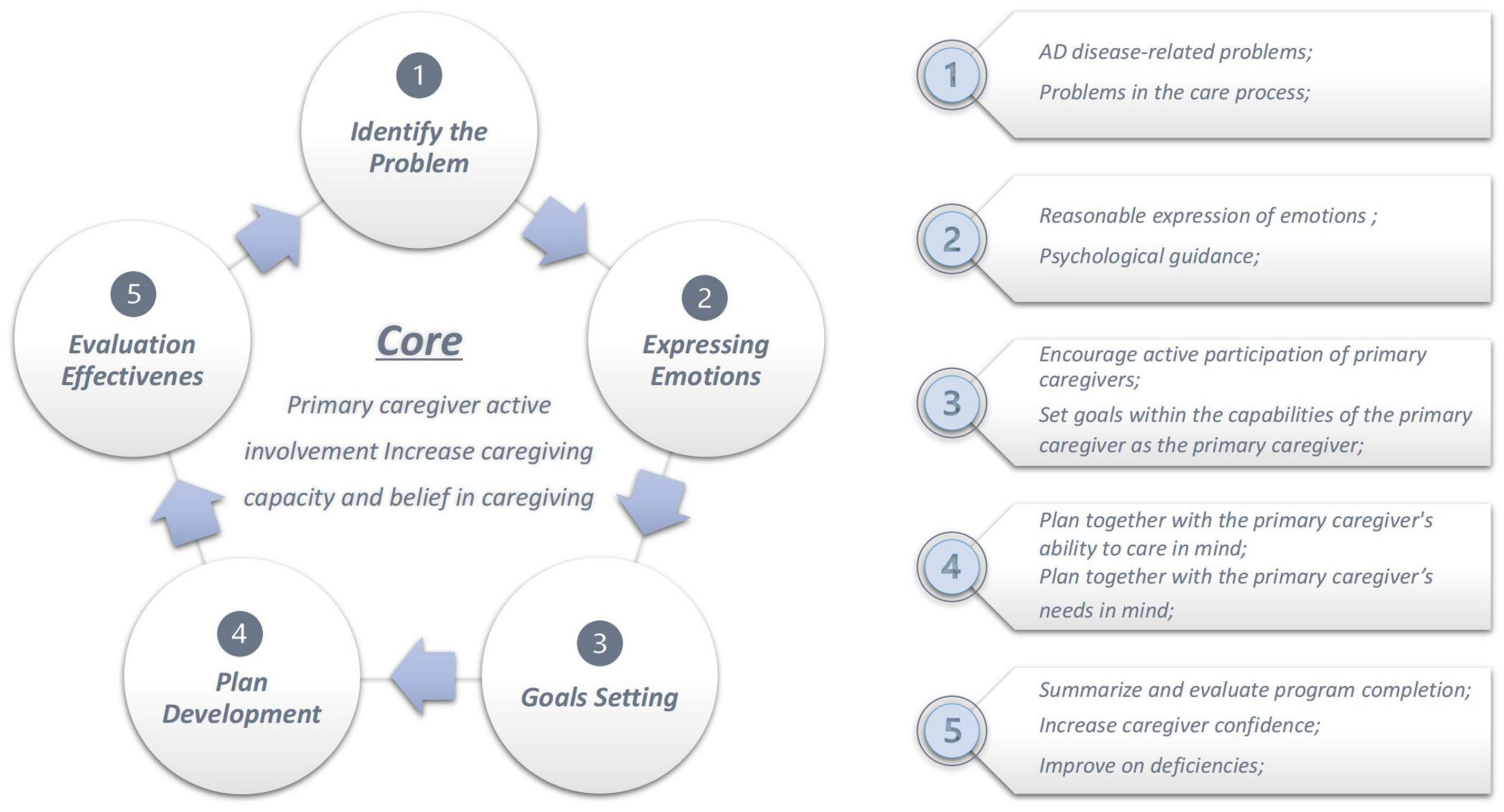
Empowering education implementation flow chart.

**Figure 2:**
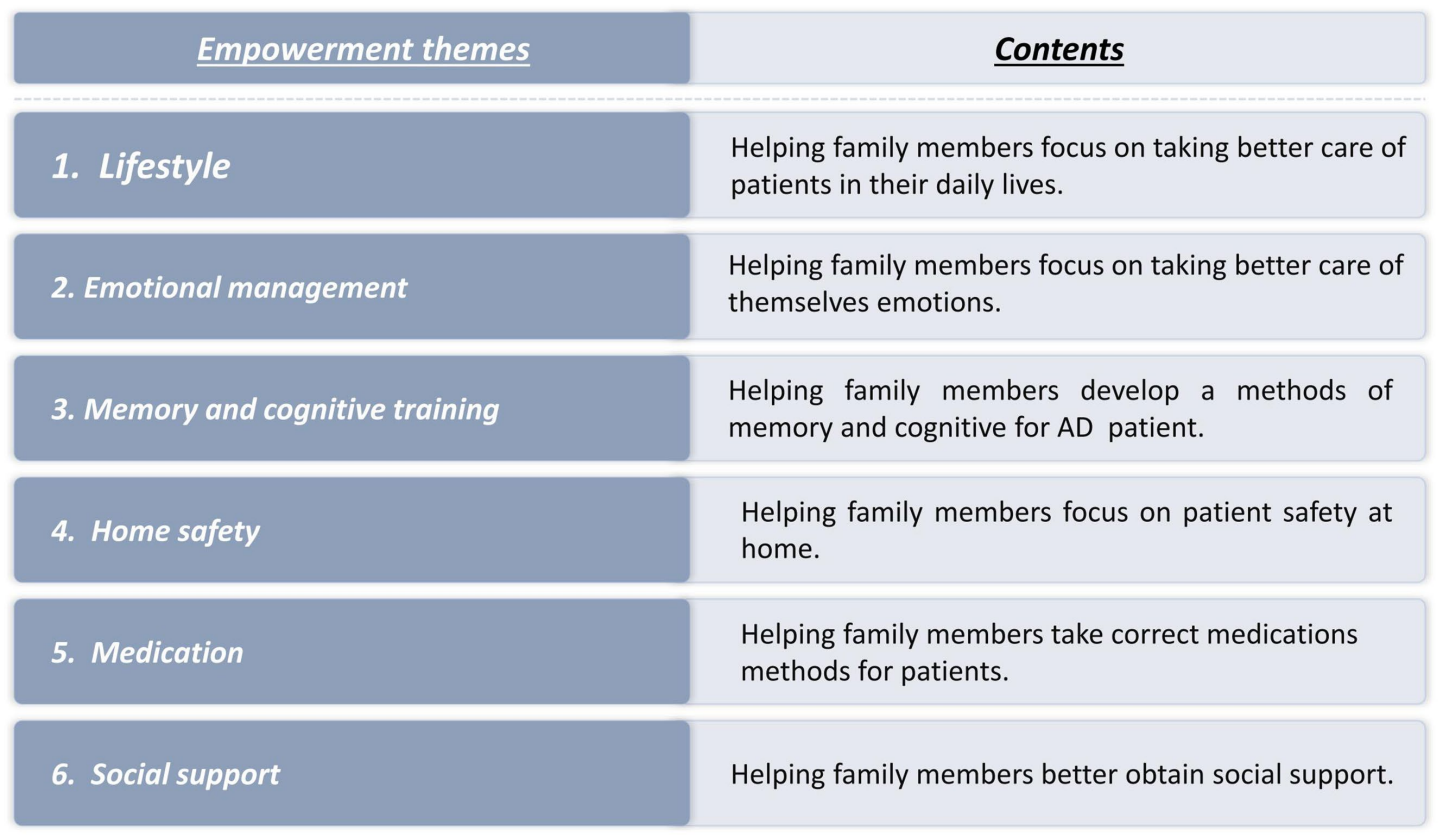
Empowerment healthy education program.

The intervention group participated in a 12-week, one-to-one program comprising six sessions lasting 45–60 min each. The program focused on empowerment education and covered six key themes: (1) Lifestyle empowerment; (2) Emotion management; (3) memory cognitive training methods for AD patients; (4) home safety; (5) Medication management; and (6) Social support empowerment. The intervention details are illustrated in Figures 1, 2 and Table 2.

**Table 2:** Details of empowerment healthy education.

Number	Themes	Content	Durations	Implementer
1	Lifestyle	Establishment problems: assess the caregiver disease-related knowledge of AD patients and encourage them to establish care problems. Express emotions: encourage caregivers to express their problems and confusion in the process of caring for the patient. Setting goals: setting goals together with caregiver based on the patient’s existing problems. Developing a plan: the researcher will provide professional caregiving skills and work with the caregivers to set competent care goals. Effectiveness evaluation: before the next intervention, the effectiveness of the caregivers’ knowledge of life care was evaluated.	30–60 min	Researcher
2	Emotional management	Establish problem: guided caregiver to summarize emotion problems that exists in the patient’s implementation. Expressing emotions: encourage caregivers to express their concerns and fears during the caregiving process. Setting goals: caregiver can build up confidence in caring for the patient and to cope with the problems and negative emotions. Developing a plan: develop a feasible emotion management plan together with the caregiver, researcher providing scientific support to the caregiver. Effectiveness evaluation: caregiver’s emotion was evaluated by researcher and psychological consultant.	30–60 min	Researcher, psychological consultant.
3	Memory and cognitive training	Establishing problem: guided caregivers to establishing questions about memory and cognitive training methods for people with AD. Expressing emotions: caregiver express confusion about memory and cognitive training for AD patients. Setting goals: to be able to acquire cognitive training methods for people with AD Developing a plan: make a plan for memory training with the caregiver and provide professional support, such as digital stimulation memory rehabilitation method. Effectiveness evaluation: caregiver correctly grasped the method of memory cognitive training.	30–60 min	Researcher
4	Home safety	Establishing problems: guided caregiver to considering how to ensure the patient’s safety at home. Expressing emotions: encourage caregiver talk about concerns of patient’s safety at home. Setting goals: caregivers can grasp the safety hazards and to prevent the safety hazards. Developing a plan: encourage caregivers to actively participate in home safety management programs. For example, prevention of wandering, falls, etc. Effectiveness evaluation: patients did not slip, fall, get injured, or lose their way.	30–60 min	Researcher
5	Medication	Establish problem: guide caregiver express question about the patient’s medication. Expressing emotions: encourage caregivers to talk about their questions about the patient’s medication. Setting goals: caregivers familiarize with medication safety, and adverse drug reactions. Developing a plan: the researcher sends the knowledge related to medication safety through face-to-face and WeChat group; e.g., in order to avoid missing and wrong medication, caregivers must accompany the AD patients and wait until they take all the medication before they leave; for patients with swallowing disorders, it is necessary to crush the medicine and dissolve it in water or mix it with food; carefully observe the patient’s reaction after taking the medicine, such as choking, coughing and adverse drug reaction; at the same time, place the medicine safely. Effectiveness evaluation: caregivers learn about medication safety.	30–60 min	Researcher, clinician
6	Social support	Establishing problem: encouraging caregiver to express need for social support. Expressing emotions: encouraging caregivers to release adverse emotions such as anxiety and depression caused by the burden of caring for the patient. Setting goals: caregivers were able to take the initiative to seek help from family, medical and social support. Developing a plan: researcher supported the caregivers with professional information by setting up a WeChat group; encouraging caregivers to share methods and communicate with their family members and friends. Organizing free offline clinics and Tencent conferences with disease-related knowledge lecture. Effectiveness evaluation: during follow-up, assess whether the caregivers will actively seek support, and build up confidence in being able to take care of the patients in the long term.	WeChat group	Researchers, nurses, doctors

Delivery of the program involved a combination of group sessions, individual counseling, and the provision of a health manual. Additionally, a WeChat group was created to offer free real-time guidance and Q&A available 24 h a day to all participants. Follow-up visits were conducted every 2 weeks via WeChat or telephone to assess participants’ understanding of the health education material and to address caregivers’ emotions while reinforcing caregiving beliefs.

To ensure standardized delivery empowering health education, the intervention program was reviewed by an expert panel comprising dementia specialists, nurses with over 3 years of experience in dementia care, and psychological counselors. The researchers who provided the empowering health education had undergone relevant training.

### 2.7 Measurements

The primary outcome measures included the knowledge level of dementia, assessed using the Dementia Knowledge Assessment Scale (DKAS), and caregiver readiness, measured by the Caregiver Preparedness Scale (CPS). Additionally, secondary outcomes comprised psychological evaluations using the Hospital Anxiety and Depression Scale (HADS) and Positive Aspects of Caregiving (PAC).

The DKAS, developed by Australian academic Annear et al. (41) in 2015, is suitable for assessing health service workers or family caregivers lacking formal dementia training. It comprises four subscales with 25 items covering dementia etiology, characteristics, communication and behavior, caregiving considerations, risks, and health promotion. Each item offers five response options of “correct, probably correct, incorrect, probably incorrect, unknown,” scored from 0 to 2, with higher scores indicating better caregiver knowledge. The Cronbach’s alpha coefficient is 0.818.

The CPS, developed by Archbold et al. (42), assesses the readiness of informal caregivers using a 5-point Likert scale ranging from 0 to 4 indicating “not at all ready to fully ready.” Higher scores indicate better readiness for care. The Cronbach’s α coefficient was 0.925.

The PAC scale was developed by Tarlow et al. measures caregivers’ positive feelings (43), using nine items across two dimensions: self-affirmation and outlook on life. Each item is rated on a 5-point Likert scale, ranging from 1 to 5 on a scale from “strongly disagree” to “strongly agree,” with a total of 45 points. Higher total scores reflect a more positive perception of caregiving. The Cronbach’s α coefficient was 0.903.

The HADS, developed by Zigmond et al. (44), assesses anxiety and depression through two dimensions, HA and HD, each comprising seven items. Items are scored from 0 to 3 points, with higher scores indicating more severe symptoms. The Cronbach’s alpha coefficients of HA and HD dimensions were 0.834 and 0.810, respectively.

### 2.8 Ethical approval and study registration

Ethical approval for this study was obtained from the ethics committee of The First Central Hospital of Tianjin (ethics number: 2021N166KY). The trial was registered under the Chinese Clinical Trial Registry, ChiCTR (ChiCT2300071777). Due to processing time, the registration was completed after the recruitment of the first participant.

### 2.9 Increase adherence to the intervention

To increase participant engagement and reduce dropout rates during the study, the following measures were implemented:

1. Establishing a good relationship with participants to gain trust and support.
2. Creating a WeChat group, where the researcher could provide free assistance with difficulties and emergencies encountered during the caregiving process.
3. Preventing participant dropout due to fatigue.
4. Providing gifts after completing the questionnaire everyone each intervention day. The type and number of gifts were consistent for all participants to eliminate any influence on the outcomes (e.g., pill cutters, small pill boxes).

### 2.10 Statistical analysis

Data were entered, managed, and analyzed using IBM SPSS Statistics version 21.0. General data for both groups were expressed as percentages (%), while measurement data were presented as mean ± standard deviation. Normality tests were conducted before data comparison and analysis. Independent samples *t*-tests were used to investigate DKAS, CPS, PAC, and HADS scores for the intervention and control groups at three time points: pre-intervention (T0), 4 weeks post-intervention (T1), and 12 weeks post-intervention (T2). Chi-square tests were employed to assess balance in count data, and repeated measures ANOVA was used to analyze changes in indicators between the control and intervention groups over time.

## 3 Results

### 3.1 Characteristics of the participants

**Figure 3:**
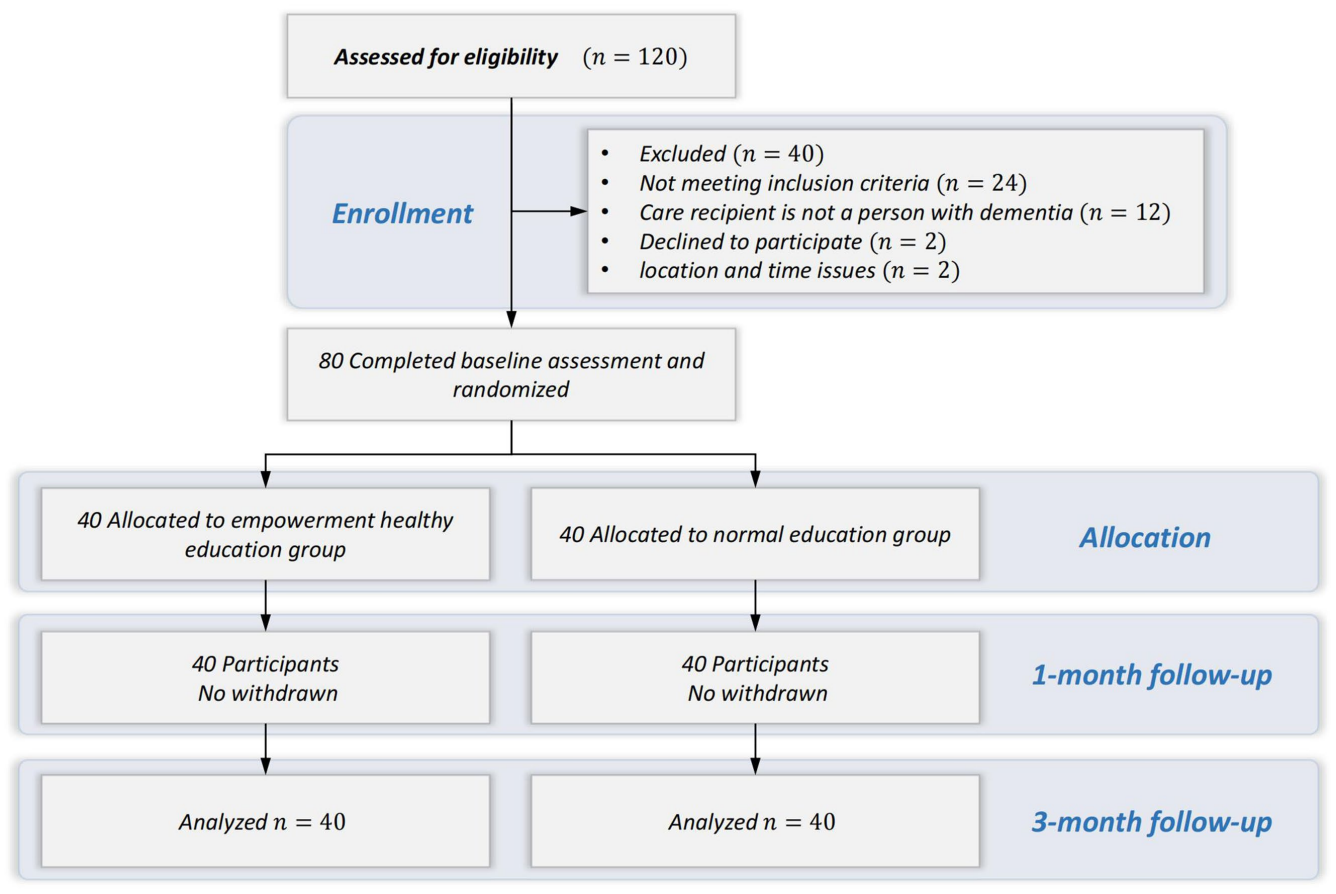
Consort flow chart. Changes in primary and secondary outcomes.

One hundred and twenty family caregivers expressed interest in participating in this study. Of these, 96 met the sample selection criteria and 80 agreed to participate in the study. The CONSORT flowchart is shown in Figure 3. The demographic of the family caregivers is summarized in Table 3. Most participants were female (60%), with a mean age of 56.97 (S.D. = 13.97) years. The majority were children of patients with dementia (47.5%), and provided care for more than 12 h per day (63.7%). There were no statistically significant differences in baseline data between the two groups (*p* > 0.05).

**Table 3:** Characteristics of the participants.

	ALL (%)	IG	CG	t/X^2^	*p* value
Gender				3.200	0.074
Male	32 (40)	16	16		
Female	48 (60)	24	24		
Age				−1.401	0.165
Mean (SD)	56.97 ± 13.97	59.15 ± 14.21	54.8 ± 13.55		
Relationship				−0.145	0.885
Spouse	34 (42.5)	13	21		
Son/Daughter	38 (47.5)	26	12		
Son/Daughter in law	6 (7.4)	1	5		
Sibling	1 (1.3)	0	1		
Friend	1 (1.3)	0	1		
Income per month				−0.317	0.752
Less than 1,000	2 (2.5)	0	2		
1,001 ~ 3,000	4 (5.0)	3	1		
3,001 ~ 5,000	36 (45.0)	20	16		
More than 5,001	38 (47.5)	17	21		
Education level				0.433	0.660
Primary	14 (17.5)	6	8		
Secondary	21 (26.2)	11	10		
Tertiary above	45 (56.3)	23	22		
Employment status				−0.739	0.462
Employed	30 (37.5)	17	13		
Retired/unemployed	44 (55.0)	20	24		
Other	6 (7.5)	3	3		
Duration of care (Month)				−0.626	0.533
3 ~ 6 h	22 (27.5)	12	10		
7 ~ 12 h	7 (8.8)	4	3		
More than 12 h	51 (63.7)	24	27		
Duration of care per week (Month)				−0.626	0.225
3 ~ 4 days	17 (21.2)	11	6		
5 ~ 6 days	3 (3.8)	1	2		
7 days	60 (75.0)	28	32		
Self-awareness of physical condition				0.548	0.585
Very bad	2 (2.5)	2	0		
Bad	2 (2.5)	1	1		
General	27 (33.8)	11	16		
Good	40 (50.0)	23	17		
Very good	9 (11.2)	3	6		
Self-awareness of financial stress				0.420	0.676
No pressure	54 (67.5)	26	28		
Yes, affordable	24 (30.0)	13	11		
Yes, it’s hard to afford	2 (2.5)	1	1		

IG, Intervention Group; CG, Control Group; CPS, Caregiver Preparedness Scale; HA, Anxiety; HD, Depression; DKAS, Dementia Knowledge Assessment Scale.

### 3.2 Baseline comparison

An independent samples *t*-test was used to analyze dementia knowledge, caregiver readiness, self-affirmation, life outlook, anxiety, depression in the two groups before the intervention. The results shown in Table 4, indicate no statistically significant differences between the two groups (*p* > 0.05), demonstrating that the groups were comparable.

**Table 4:** Pre-intervention baseline comparisons ( x±s ).

Instrument	IG (*n* = 40)	CG (*n* = 40)	*t*	*p*
DKAS	22.77 ± 6.38	21.65 ± 7.69	0.711	0.479
CPS	19.10 ± 7.08	20.57 ± 5.86	−1.014	0.314
Self-affirmation	18.02 ± 3.08	18.02 ± 3.08	1.685	0.096
Life outlook	14.10 ± 3.49	14.90 ± 3.32	−1.049	0.297
HA	5.95 ± 3.23	4.92 ± 2.47	1.592	0.115
HD	5.57 ± 3.63	4.67 ± 2.72	1.253	0.214

IG, Intervention Group; CG, Control Group; DKAS, Dementia Knowledge Assessment Scale; CPS, Caregiver Preparedness Scale; HA, Anxiety; HD, Depression.

### 3.3 Changes in primary outcomes, dementia knowledge

**Figure 4:**
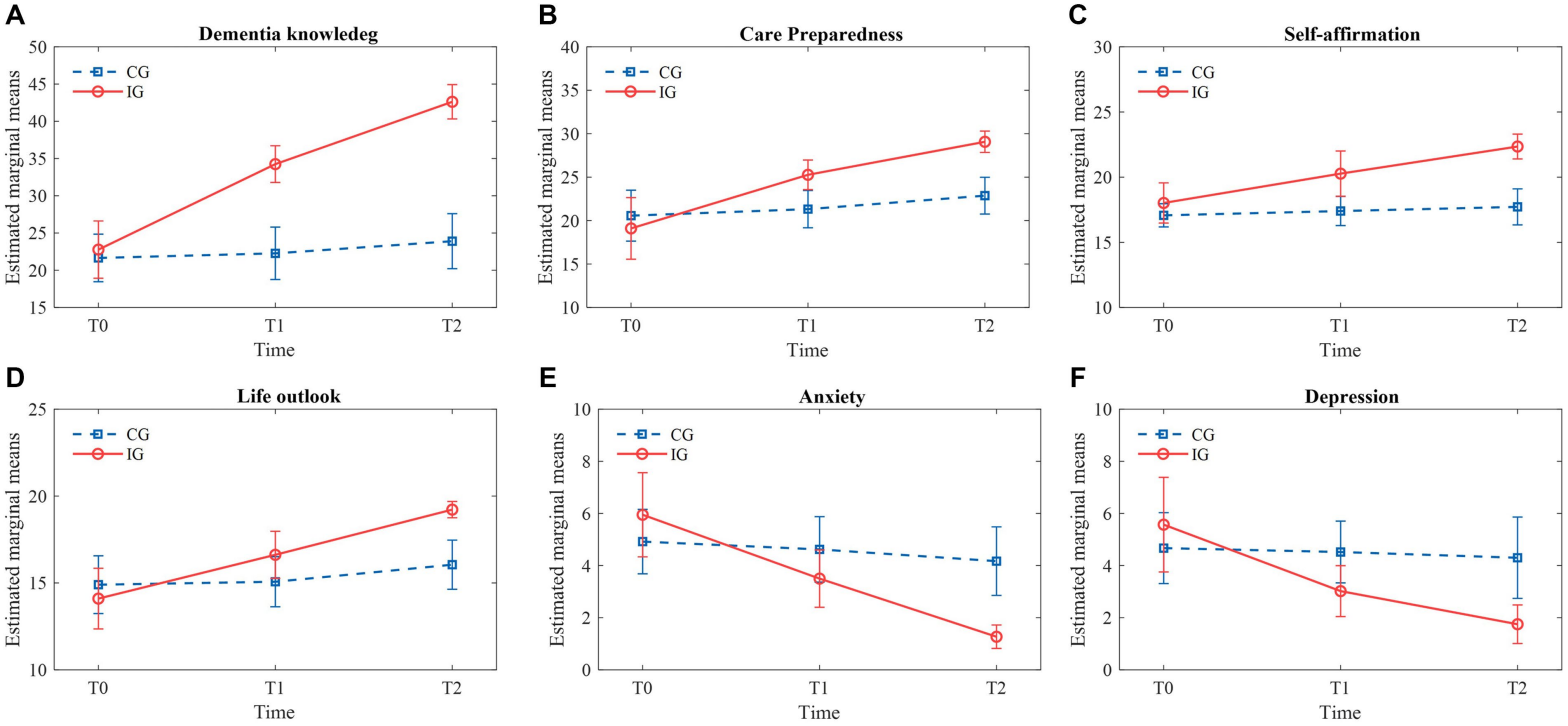
Changes in primary and secondary outcomes.

The results indicated that dementia knowledge scores of caregivers in the intervention group (IG) significantly increased compared to the control group (CG) after 4 weeks and 12 weeks (*p* < 0.05). Specifically, IG scores rose from approximately 25 at T0 to 35 at T1 and 45 at T2, marking an 80% increase over 12 weeks. In contrast, CG scores showed minimal changes from about 24 at T0 to 25 at T1 and 26 at T2, representing only an 8.3% increase. The main effect of the grouping factor was statistically significant (*F* = 78.643, *p* < 0.001), as was the main effect of the time factor (*F* = 125.216, *p* < 0.001). The significant interaction effect between the time factor and the grouping factor (*F* = 80.609, *p* < 0.001) suggests differential changes over time between the groups (Table 5). As illustrated in Figure 4A, the trend of increasing dementia knowledge scores was significantly more pronounced in the IG, underscoring the intervention’s effectiveness.

**Table 5:** Data analysis on study outcomes and effect sizes (Between groups).

		IG (*n* = 40)		CG (*n* = 40)		95% confidence intervals (pre-post-test mean difference)	Mann- Whitney’s test for between-group comparisons mean difference			Repeat measurement analysis (*F/P/* η )		
Instrument	Time	*M*	*SD*	*M*	*SD*	Lower and upper limits	*t*	*p* value	Effect size (*d*)	Time effect	Group effect	Time*Group effect
DKAS	Pre	22.77	6.38	21.65	7.69	−2.02, 4.27				125.216/<0.001/0.616	78.643/<0.001/0.502	80.609/<0.001/0.508
	Post	34.25	7.04	22.27	4.93	9.26, 14.68	8.808	<0.001	6.08			
	Follow-up	42.62	7.39	23.90	4.61	15.98, 21.46	13.587	<0.001	6.16			
CPS	Pre	19.10	7.08	20.57	5.86	4.37, 1.42				95.842/<0.001/0.551	9.357/<0.05/0.336	39.390/<0.001/0.107
	Post	25.27	3.41	21.32	4.30	2.21, 5.68	4.544	<0.001	3.88			
	Follow-up	29.07	2.45	22.87	4.23	4.66, 7.73	8.016	<0.001	3.45			
Self-affirmation	Pre	18.02	3.08	17.07	1.78	−0.17, 2.07				39.477/<0.001/0.336	33.355/<0.001/0.216	21.549/<0.001/0.300
	Post	20.27	3.47	17.40	2.23	1.57, 4.17	4.402	<0.001	2.92			
	Follow-up	22.35	1.91	17.72	2.76	3.56, 5.68	8.698	<0.001	2.37			
Life outlook	Pre	14.10	3.49	14.90	3.32	−2.31, 0.71				67.998/<0.001/0.466	5.667/<0.05/0.260	27.411/<0.001/0.068
	Post	16.62	2.71	15.07	2.88	0.30, 2.79	2.474	<0.05	2.80			
	Follow-up	19.22	0.94	16.05	2.83	2.23, 4.11	6.714	<0.001	2.11			
HA	Pre	5.95	3.23	4.92	2.47	−0.25, 2.30				95.199/<0.001/0.550	4.081/<0.05/0.391	49.981/<0.001/0.050
	Post	3.50	2.20	4.62	2.51	−2.17, −0.07	−2.125	<0.05	2.36			
	Follow-up	1.27	0.90	4.17	2.63	−3.22, −1.22	−6.572	<0.001	1.97			
HD	Pre	5.57	3.63	4.67	2.72	−0.52, 2.32				31.950/<0.001/0.291	4.310/<0.05/0.220	22.058/<0.001/0.052
	Post	3.02	1.95	4.52	2.37	−2.46, −0.53	−3.084	<0.05	2.17			
	Follow-up	1.75	1.48	4.30	3.12	−3.30, −0.99	−4.666	<0.001	2.44			

IG, Intervention Group; CG, Control Group; DKAS, Dementia Knowledge Assessment Scale; CPS, Caregiver Preparedness Scale; HA, Anxiety; HD, Depression.

### 3.4 Changes in primary outcomes, care readiness

At 4 weeks and 12 weeks post-intervention, caregiver preparedness in the IG significantly increased compared to the CG (*p* < 0.05). IG scores improved from about 20 at T0 to 25 at T1 and 30 at T2, reflecting a 50% increase over 12 weeks. In contrast, CG scores exhibited only slight changes from around 21 at T0 to 22 at T1 and 23 at T2, indicating a 9.5% increase. The main effect of the grouping factor was statistically significant (*F* = 9.357, *p* < 0.05), as was the main effect of the time factor (*F* = 95.842, *p* < 0.001). The significant interaction effect between the time factor and the grouping factor (*F* = 39.390, *p* < 0.001) demonstrates differing trends over time between the groups (Table 5). Figure 4B shows a notable upward trend in caregiving preparedness scores in the IG, highlighting the substantial impact of the intervention.

### 3.5 Changes in secondary outcomes, self-affirmation

Self-affirmation scores in the IG significantly increased at 4 weeks and 12 weeks post-intervention compared to the CG (*p* < 0.05). IG scores rose from approximately 15 at T0 to 18 at T1 and 21 at T2, indicating a 40% increase. In contrast, CG scores remained relatively stable, changing from around 16 at T0 to 16.5 at T1 and 17 at T2, representing a 6.3% increase. The main effect of the grouping factor was statistically significant (*F* = 33.355, *p* < 0.001), as was the main effect of the time factor (*F* = 39.477, *p* < 0.001). The significant interaction effect between the time factor and the grouping factor (*F* = 21.549, *p* < 0.001) underscores the differing trends over time between the groups (Table 5). Figure 4C demonstrates a significant upward trend in self-affirmation scores in the IG, reinforcing the intervention’s efficacy.

### 3.6 Changes in secondary outcomes, life outlook

Life outlook scores in the IG significantly improved at 4 weeks and 12 weeks post-intervention compared to the CG (*p* < 0.05). IG scores increased from about 13 at T0 to 17 at T1 and 20 at T2, representing a 53.8% increase. Meanwhile, CG scores showed a modest increase from around 13.5 at T0 to 14 at T1 and 15 at T2, an 11.1% increase. The main effect of the grouping factor was statistically significant (*F* = 5.667, *p* < 0.05), and the main effect of the time factor was highly significant (*F* = 67.998, *p* < 0.001). The significant interaction effect between the time factor and the grouping factor (*F* = 27.411, *p* < 0.001) indicates differing trends over time between the groups (Table 5). Figure 4D clearly illustrates the more significant upward trend in life outlook scores in the IG, validating the intervention’s positive impact.

### 3.7 Changes in secondary outcomes, anxiety

Anxiety scores in the IG significantly decreased compared to the CG (*p* < 0.05). IG scores dropped from about 6.5 at T0 to 4 at T1 and 2 at T2, marking a 69.2% reduction. In contrast, CG scores remained relatively stable, changing from around 5.5 at T0 to 5 at T1 and 4.5 at T2, representing an 18.2% reduction. The main effect of the grouping factor was statistically significant (*F* = 4.081, *p* < 0.05), as was the main effect of the time factor (*F* = 95.199, *p* < 0.001). The significant interaction effect between the time factor and the grouping factor (*F* = 49.981, *p* < 0.001) highlights the differing changes over time between the groups (Table 5). Figure 4E shows a significant downward trend in anxiety scores in the IG, demonstrating the intervention’s effectiveness in reducing anxiety.

### 3.8 Changes in secondary outcomes, depression

Depression scores in the IG significantly decreased compared to the CG (*p* < 0.05). IG scores declined from about 6.5 at T0 to 4 at T1 and 2 at T2, indicating a 69.2% reduction. Conversely, CG scores changed from around 6.5 at T0 to 6 at T1 and 5.5 at T2, representing a 15.4% reduction. The main effect of the grouping factor was statistically significant (*F* = 4.310, *p* < 0.05), as was the main effect of the time factor (*F* = 31.950, *p* < 0.001). The significant interaction effect of time and grouping (*F* = 22.058, *p* < 0.001) indicates differing trends over time between the groups (Table 5). Figure 4F highlights the more pronounced downward trend in depression scores in the IG, further supporting the intervention’s substantial impact.

### 3.9 Adverse events

No study-related adverse events were reported in either the cognitive training or active control groups.

## 4 Discussion

This trial investigated the impact and effectiveness of an empowerment theory-based approach to health education in improving dementia knowledge, caregiver readiness, positive aspects of care, and reducing anxiety and depression among dementia caregivers. The summarized results indicate that health education based on empowerment theory is highly effective. Through this approach, caregivers significantly enhanced their caregiving competencies and beliefs, enabling them to cope with the challenges associated with Alzheimer’s disease and to adapt more effectively to care needs at both individual and family levels.

Regarding the primary outcomes, we found that empowerment theory-based health education led to a significant improvement in dementia knowledge, as measured by the DKAS and caregiver preparedness as measured by the CPS, compared with those in the control group. The results highlight that the knowledge of Alzheimer’s disease among informal caregivers in China is low, with caregivers expressing an urgent need for specialized health education from medical professionals to enhance their understanding of the disease (45).

Due to medical and economic constraints, most Alzheimer’s disease patients in China are cared for at home by family members. As the disease progresses, patients experience a gradual decline in cognition and self-care abilities, making family caregivers crucial for their survival. Therefore, it is essential for these caregivers to be well-versed Alzheimer’s disease-related knowledge. Similar studies on family caregivers of children with heart disease (36) have shown that empowerment interventions improve caregivers’ predisposition and readiness. This finding was corroborated in our study.

Empowerment education facilitates effective interactions with caregivers. Notably, even the control group, which received conventional health education, showed improvements in dementia knowledge and caregiver preparedness during the follow-up periods, although these improvements were less significant.

Caregiving readiness refers to the preparedness of caregivers to meet all aspects of the care recipient’s needs and emergencies, impacting both the quality of care and the caregiver quality of life (42). Empowerment helps caregivers recognize current situations and potential needs, improve their negative emotions, enhance their ability to make autonomous decisions, solve problems, and improve interpersonal communication skills. In short, empowerment stimulates caregiver’s potential, fostering a sense of self-efficacy and control over their lives (36). This readiness is crucial to the quality of life and disease prognosis of patients.

According to the theory of empowerment, health education plays a crucial role in caregivers of AD patients. Caregivers require cognitive knowledge to effectively address challenges and difficulties in caregiving. Through effective interaction and empowering education caregivers are encouraged to express their concerns and difficulties. We offer understandable teachings on AD management using various intervention methods. Caregivers with higher educational levels tend to be more receptive and willing to learn new knowledge. Using online lectures, free consultations, and Q&A sessions helps enhance knowledge and confidence among caregivers.

Empowering education equips caregivers with essential Alzheimer’s disease-related knowledge and caregiving skills, ultimately improving their satisfaction with the care they provide. By encouraging caregivers to identify and express issues during caregiving, empowering health education enables them to set care goals, develop improvement plans, and implement effective solutions. Regular evaluations reinforce positive impacts and enhance self-confidence. This theory-based intervention strengthens caregivers’ understanding of Alzheimer’s disease-related diseases, emotional management, and caregiving skills, thereby enhancing their overall preparedness. Researchers communicate with equality and respect, fostering trust and openness with caregivers. As caregivers express their anxieties, relationships improve, and they are more likely to seek support, leading to a reduction in caregiving stress. Therefore, theory-based empowering health education significantly improves caregivers’ preparedness for proving care to AD patients.

For secondary outcomes, our findings indicate that empowerment theory-based health education demonstrates significant effectiveness in improving self-affirmation and life outlook, as measured by the PAC, as well as reducing anxiety and depression, measured by the HADS, compared with those in the control group. This trend aligns with the primary outcomes of dementia knowledge and caregiver preparedness.

Firstly, empowerment-based health education enables caregivers to enhance their self-recognition and self-confidence by providing relevant knowledge and skills. The study results reveal that the intervention group exhibited significantly higher self-affirmation scores than the control group, indicating that empowerment theory-based health education intervention can improve caregivers’ self-affirmation levels. This underscores the importance of providing accessible and understandable information, as along with continuous learning and training, to effectively help caregivers comprehend relevant AD knowledge, thereby enhancing their caregiving abilities and the quality of care they provide.

Secondly, empowerment-based health education positively influences caregivers’ life outlook levels by using various intervention methods, fostering an optimistic attitude toward their future lives. The results suggest that this approach can significantly improve caregivers’ outlook on life and empower them to establish cognitive awareness and a sense of control over the disease, which align with a previous study (46). Among these, anxiety, characterized by constant tension, worry, apprehension, and uneasiness about potential adverse consequences or ambiguous threats (47), is particularly prevalent among caregivers of AD patients who often face physical and psychological burdens, sometimes leading to mood disorders such as anxiety and depression. As mentioned earlier, the psychological well-being of family caregivers plays a crucial role in both the quality of life of patients and disease progression (24, 25).

Research has demonstrated that family caregivers who lack understanding of patient treatment and prognosis, struggle to anticipate disease progression and lose confidence in recovery are susceptible to negative emotions such as anxiety and depression (48). Additionally, when family caregivers lack robust social support, their caregiving burden significantly intensifies. Empowerment-based health education aims to actively listen and support caregivers’ emotional needs, offer professional knowledge and caregiving skills training, and establish effective social support networks. These interventions effectively alleviate caregivers’ psychological stress and enhance their mental well-being.

Empowerment education is particularly useful because it addresses the psychological and emotional needs of caregivers, providing them with sustainable skills to manage ongoing caregiving challenges. Unlike conventional approach that may offer static information, empowerment education promotes continuous growth and adaptability, which are essential for long-term caregiving success. Future research should focus on integrating digital tools to provide flexible learning options, conducting long-term studies to evaluate sustained impacts, and adapting materials for diverse cultural contexts. By emphasizing the unique benefits of empowerment education and addressing these challenges, we aim to support and empower caregivers, ultimately improving the quality of care for people with dementia.

This study offers valuable insights into developing a health promotion model for family caregivers of AD patients. The findings underscore the significance of adopting of an empowerment-based approach to health education, which empowers family caregivers within health promotion frameworks. Given that family caregivers constitute the immediate social environment for AD patients, prioritizing the health of family caregivers becomes crucial. Both researchers and society should actively engage in monitoring the health status of caregivers and provide relevant medical services and support to effectively address their caregiving needs. Moreover, specialized caregiver programs tailored to specific conditions will be essential, particularly for other types of AD such as PCA and lvPPA. These variants, often caused by underlying AD pathology, present unique challenges characterized by cortical visual and/or linguistic impairments, with relatively spared memory function. As such tailored support programs are essential to address the distinct needs of caregivers facing these challenges (49).

### 4.1 Limitations

Several limitations need to be acknowledged in this study. Firstly, the research was conducted at a single hospital in Tianjin, which may restrict its applicability to other settings or populations with diverse cultural backgrounds or healthcare systems. The prevalence of media usage, particularly WeChat in Chian, could have significantly influenced our results. However, this prevalence may vary in other regions, necessitating consideration of media prevalence in future studies. Secondly, although we used validated scales to measure various aspects of caregiving outcomes, self-report measures are susceptible to social desirability bias or recall bias. Thirdly, we did not assess the long-term effects of the intervention beyond the 12-week follow-up period. Lastly, this is a single-blind study, and non-blinded interventionists may have influenced the results. We recommend that future research investigate the sustainability of intervention effects over an extended period.

## 5 Conclusion

In conclusion, healthy education grounded in empowerment theory significantly improved caregivers’ understanding of dementia, their readiness for caregiving, their positive outlook for caregiving, and reduced their levels of anxiety and depression when caring for AD patients. It is imperative for medical professionals to continuously update their knowledge to cater to the diverse needs of AD caregivers, with follow-up support extended as necessary. Early diagnosis and intervention are vital in slowing disease progression, lessening the burden on family caregivers, and ultimately improving the quality of life of both patients and caregivers.

## Data Availability

The raw data supporting the conclusions of this article will be made available by the authors, without undue reservation.
